# Mechanical Disintegration and Particle Size Sieving of *Chondrus crispus* (Irish Moss) Gametophytes and Their Effect on Carrageenan and Phycoerythrin Extraction

**DOI:** 10.3390/foods10122928

**Published:** 2021-11-26

**Authors:** Adiguna Bahari, Katlijn Moelants, Marie Kloeck, Joel Wallecan, Gino Mangiante, Jacques Mazoyer, Marc Hendrickx, Tara Grauwet

**Affiliations:** 1 Global Core R&D, Cargill R&D Centre Europe, Havenstraat 84, 1800 Vilvoorde, Belgium; Adiguna_Bahari@cargill.com (A.B.); Joel_Wallecan@cargill.com (J.W.); 2 Laboratory of Food Technology, Department of Microbial and Molecular Systems, KU Leuven, Kasteelpark Arenberg 22 Box 2457, 3001 Leuven, Belgium; mariekloeck@gmail.com (M.K.); marceg.hendrickx@kuleuven.be (M.H.); tara.grauwet@kuleuven.be (T.G.); 3 Product & Processes Development Centre, Cargill Starches Sweeteners & Texturizers, 50500 Carentan, France; Gino_Mangiante@cargill.com (G.M.); Jacques_Mazoyer@cargill.com (J.M.)

**Keywords:** particle size reduction, hybrid carrageenan, valorization, phycobiliproteins, phycoerythrin, red seaweed, surface area, cuticle

## Abstract

To better understand the migration properties of hybrid carrageenan from the seaweed tissue during carrageenan extraction, the effect of increasing the seaweed surface area by the mechanical disintegration of gametophyte *Chondrus crispus* chips was studied under various temperature and time extraction conditions. Dried *Chondrus crispus* seaweed chips were milled by a rotor beater mill and classified into eight different size fractions by sieving with varying mesh sizes from 50 to 2000 μm. During extraction at 22 °C, the red color of the filtrate increased significantly with the decreasing particle size of the fraction, correlating with the increasing phycoerythrin concentration (from 0.26 mg PE/g dry seaweed in the >2000 μm size fraction to 2.30 mg PE/g dry seaweed in the <50 μm size fraction). On the other hand, under the same extraction conditions, only a small increase in carrageenan precipitate was obtained with the decreasing size fractions (from no recovery in the >2000 μm size fraction to 2.1 ± 0.1 g/kg filtrate in the <50 μm size fraction). This yield was significantly lower than the ones from extractions at 45 °C (5.4 ± 0.1 g/kg) or at 90 °C (9.9 ± 2.1 g/kg) for the same particle size and time conditions. It could be concluded that hybrid carrageenan extraction is not surface area dependent, while phycoerythrin is. Therefore, it seems that phycoerythrin and carrageenan extraction follow different mechanisms. This creates potential for the selective extraction of each of those two compounds.

## 1. Introduction

Red macroalgae (Rhodophyta) carrageenophytes are the most farmed type of seaweed in the world. In 2017, they contributed to around 40% of global seaweed production [1]. Carrageenophytes are harvested for their carrageenan, a sulfated galactan widely used in food applications for gelling and/or thickening properties. The classical industrial production of carrageenan is relatively straightforward: Seaweed is heated at high temperature with water, and alkali is used to enhance carrageenan’s gelling strength [2]. Through this process, carrageenan is effectively solubilized and could then be recovered through filtration and isopropyl alcohol (IPA) precipitation. The remaining seaweed residue however, is often denatured, limiting the studies of biochemical and biological properties of its potential valuable compounds [3].

Despite the extensive research on the carrageenan structure in the solution, little is known about how this polymer is structured and organized, relative to the other components, in the seaweed cell wall matrix. Aside from carrageenan, minor amounts of carbohydrate polymers (e.g., glucomannans, sulfated glucans) [4] and low molecular weight molecules (e.g., 6-*O*-methyl β-D-galactose residues, sulfated and non-sulfated β-D-galactose residues) [5] are also present in the cell wall of red seaweeds. In addition, red seaweeds are generally richer in proteins compared to green or brown seaweeds [6]. Coomassie brilliant blue, which is used for protein staining in the carrageenophyte *Chondracanthus teedei,* showed a strong interaction inside the cells and in the outermost cell wall layer (cuticles) near the surface of the seaweed, but not in the cell wall matrix surrounding the cells [7]. A study that isolated the cell wall matrix of *Neopyropia tenera* (formerly *Porphyra tenera*) found that proteins only amount to 2.4–3.4 wt% of the cell wall mass. Meanwhile, in the cuticular region, the protein content amounts to 37.6–44.4 wt% of the cuticular mass [8]. Since carrageenan is also a main component of the cuticle layer, it is reasonable to think that carrageenan could form strong bonds with the cuticular proteins due to the carrageenan’s polyanionic nature [9]. This interaction between carrageenan and the cuticular proteins is believed to be a contributing factor in keeping the physical integrity of the cuticle layer [8].

Inside the seaweed cells and away from the cell wall matrix, pigment proteins called the phycobiliproteins exist in the cytoplasmic surface of the thylakoid membrane [10]. Phycobiliproteins represent a family of fluorescent proteins unique to red seaweed and cyanobacteria that serves as a photosynthetic light-harvesting antenna complex. Phycobiliproteins are connected to each other via linker polypeptides, making a macromolecular structure (phycobilisome) that is covalently bound to the photosystem core in the membrane [11,12,13]. When the cells are lysed or broken, the phycobilisome dissociates into water soluble complexes and imparts color to the solvent [10]. The colors of these proteins arise from the linear tetrapyrroles (bilins) that are covalently bound to the proteins through a thioether bond cysteinyl residue [14]. The red colored phycoerythrin (PE) (absorption around 490 and 540–567 nm) is the most abundant phycobiliprotein in red seaweeds [10]. PE is valuable, and often sourced from Cyanobacteria or microalgae for use in molecular biology applications [10] as well as foods [15] and cosmetics [16].

Different ideas have been proposed to make more value out of red seaweeds and valorization of other compounds, such as the phycobiliproteins. A biorefinery approach, through implementing additional steps before or after the carrageenan extraction, seems a promising way forward [17]. The use of enzymes to separate seaweed proteins and carrageenan has also been proposed [18]. An equally important approach is to understand the role of each extraction parameter and how it affects the carrageenan extraction. In a recent study, our group described how processing parameters, such as temperature, time, and medium conditions, affected hybrid carrageenan (comprising of kappa, iota, and their precursor units) extraction from the gametophyte *Chondrus crispus* chips (around 1 cm in size) [19]. It was found that hybrid carrageenan extraction is driven by high temperature during extraction and that the removal of endogenous salts improves hybrid carrageenan yield at lower extraction temperatures. The study suggested a relationship between the hybrid carrageenan’s structure and its ability or inability to leach out of the cell wall.

In addition to the extraction temperature, time, and medium conditions, the particle size of the seaweed chips could be another interesting variable to affect the extraction of hybrid carrageenan. Smaller seaweed particle sizes would have a larger specific surface area (surface area-to-mass ratio), which could lead to an increase in hybrid carrageenan extraction yield at milder temperatures.

In this research, the effect of particle size on hybrid carrageenan extraction was evaluated. Gametophyte *Chondrus crispus* was chosen as the seaweed source as it contains hybrid gelling carrageenan and their precursor units. The mechanical disintegration of the seaweed was achieved through rotor beater milling, followed by the obtainment of several size fractions through sieving. The microstructure, particle size distribution, and surface area of the different milled size fractions were evaluated to investigate the impact of this milling. Finally, carrageenan extraction was performed on each size fraction. The characteristics and trends of the extraction filtrate as well as the carrageenan yield extract are reported.

## 2. Materials and Methods

### 2.1. Materials

Chopped (around 1 cm in size) and dried gametophyte *Chondrus crispus* chips were obtained from selected Cargill suppliers. The reference kappa carrageenan was purchased from Fischer Scientific (Loughborough, UK). The IPA used for IPA precipitation contains ≥99.8% purity (Fischer Scientific, Loughborough, UK). All of the other reagents and solvents were of analytical grade. The hybridity of carrageenan in the gametophyte *Chondrus crispus* sample was already determined from a prior study [19].

### 2.2. Mechanical Disintegration of Seaweed Chips and Sieving It into Distinct Size Fractions

The seaweed chips (around 1 cm) were milled using a rotor beater mill SR3000 (Retsch, Derbyshire, UK) using a V-rotor and a bottom sieve of 1 mm with trapezoid holes. The milled seaweed, which was still heterogenous in size was sieved to separate them into more specific size classes. Sieves with mesh sizes 50, 100, 150, 250, 500, 1000, and 2000 μm were stacked on top of each other on an analytical sieve shaker (Retsch, Derbyshire, UK). The milled seaweed powder was sieved at an amplitude of 1.5 mm for 10 min. The powder retained on each sieve was collected resulting in size fractions of <50, 50–100, 100–150, 150–250, 250–500, 500–1000, 1000–2000, and >2000 μm.

### 2.3. Scanning Electron Microscopy (SEM) and Elemental Composition of the Seaweed Surface

Scanning electron microscopy (SEM) was carried out on each of the seaweed size fractions using a tabletop microscope TM4000 Plus II (Hitachi, Tokyo, Japan). Images were taken using a low vacuum, accelerating voltage of 15 kV, and backscattered electron imaging (BSE).

The elemental composition of the seaweed surface was visualized on the SEM images through analysis with an energy dispersive X-ray spectrometry (EDX) with Bruker Quantax 75 Xflash detector (Bruker, Billerica, MA, USA). Values were taken as the average from three different measurements on the seaweed surface.

### 2.4. Particle Size Distribution and Surface Area of the Different Size Fractions

The particle size distribution of the different size fractions was analyzed using a Mastersizer 3000 (Malvern Panalytical Ltd., Malvern, UK). For each measurement, approximately 5 g of dry powder was loaded into the feeder. The refractive and absorption indexes were set to 1.5 and 0.1, respectively. Obscuration was chosen between 1–8%. Values were taken as the average from two measurements.

The surface area of each size fraction was analyzed using a Gemini VII 2390 surface area analyzer (Micrometrics, Ottawa, ON, Canada). Approximately a third of the sample tube was filled. Samples were placed overnight in a gas adsorption preparation device (FlowPrep 060, Micrometrics, Ottawa, ON, Canada). Liquid nitrogen was used as the gaseous adsorbate. The specific surface area was calculated using the Brunauer, Emmett and Teller (BET) method.

### 2.5. Carrageenan Extraction to Obtain a Carrageenan-Rich Precipitate (CRP)

The lab-scale carrageenan extraction followed the method described in a previous study [19]. In brief, 5 g of sample (for each size fraction) was dispersed in 250 mL of demineralized water in a 500 mL glass beaker and stirred with a magnetic rod at 500 rpm for a given time (15, 30, 120 min) and temperature (22, 45, 90 °C). When a wash step is involved, the seaweed dispersion is stirred only for 30 min at room temperature (22 °C). Upon completion of the extraction, the seaweed dispersion was poured into a centrifuge jar and centrifuged at 4000× *g* for 10 min. The supernatant was passed through a 20 μm mesh sieve to obtain the final filtrate.

Carrageenan was recovered as a carrageenan-rich precipitate (CRP) by pouring an aliquot of the filtrate into 3× volume of IPA. To allow for an effective precipitation of the carrageenan, 0.3 g of KCl per 100 mL filtrate was added to the IPA solution as a precipitation aid. The CRP was then collected, manually squeezed to remove excess IPA, separated into fibers, and left to dry under a fume hood overnight. CRP was stored inside a plastic bottle and remained at room temperature away from sunlight. As no purification step was involved, the CRP could also contain non-carrageenan components (e.g., proteins).

CRP extraction was performed on each size fraction for several time and temperature combinations. Extraction temperatures of 22, 45, and 90 °C were chosen to assess the effect of room, mild, and high extraction temperatures. Extraction times of 15, 30, and 120 min were selected to investigate the potential increase in carrageenan yield over time. A previous study [19] has shown that hybrid carrageenan leaches out of the seaweed relatively quickly when heat is applied. Therefore, 15 min was chosen to evaluate the carrageenan yield at a relatively short extraction time. In addition, 120 min was selected as the extraction yield is expected to have reached a plateau. All of the extractions were performed in duplicate. All of the CRP samples underwent further drying at 105 °C for 2 h to obtain the CRP dry weight. The CRP yield was measured as a unit of concentration and calculated as follows:

(1) $$\mathrm{CRP}\;\mathrm{concentration}\;\left(\frac{g}{\mathrm{kg}}\right)=\frac{\mathrm{mass}\;\mathrm{of}\;\mathrm{dried}\;\mathrm{CRP}\;\mathrm{obtained}\;\mathrm{after}\;\mathrm{drying}\;\left(g\right)}{\mathrm{mass}\;\mathrm{of}\;\mathrm{filtrate}\;\mathrm{aliquote}\;\mathrm{used}\;\mathrm{in}\;\mathrm{IPA}\;\mathrm{precipitation}\;\left(\mathrm{kg}\right)}$$

### 2.6. Filtrate Properties

Conductivity of the extraction filtrate was measured using a SevenGo conductivity meter (Mettler Toledo, Columbus, OH, USA). The pH of the filtrate was measured using a pH meter (Knick, Lower Saxony, Germany). Red (a*) values were measured with a CIE L*a*b* space by a CM-5 spectrophotometer (Konica Minolta, Tokyo, Japan). The a* value refers to the coordinate on the red/green scale as established by the Commission Internationale de l’Eclairage (CIE). The positive a* value indicates a red color, while the negative a* value indicates a green color. Measurements were performed on each extraction replicate.

### 2.7. Phycoerythrin Analysis

The phycoerythrin (PE) content in the extraction filtrate was analyzed using a Lambda 650 UV-VIS Spectrometer (Perkin Elmer, Waltham, MA, USA), considering the absorbance/optical density (OD) at around 562 nm for PE. Prior to the spectrometry analysis, the filtrate was passed through a 0.45 μm pore size cellulose acetate syringe filter (Sartorius AG, Göttingen, Germany). The PE concentration was calculated using the equation formulated by Bennett and Bogorad [20] shown below, which also took into account the other phycobiliprotein concentrations of phycocyanin (PC) and allophycocyanin (APC):

$$\left[\mathrm{PC}\right]=\frac{{\mathrm{OD}}_{615}-0.474\left({\mathrm{OD}}_{652}\right)}{5.34}\;\;\left[\mathrm{APC}\right]=\frac{{\mathrm{OD}}_{652}-0.208\left({\mathrm{OD}}_{615}\right)}{5.09}\;\;\left[\mathrm{PE}\right]=\frac{{\mathrm{OD}}_{562}-2.41\;\left(\mathrm{PC}\right)-0.849\left(\mathrm{APC}\right)}{9.62}$$

### 2.8. Compositional Analysis of the Carrageenan-Rich Precipitates

#### 2.8.1. Sugar Analysis

Although not as specific as NMR spectroscopy to elucidate the type of carrageenan present, the sugar analysis of the carrageenan is able to give some indication on the degree of gelling units present in the carrageenan population. The main sugars in the carrageenans are galactose and 3,6-Anhydrogalactose (3,6-Angal). The precursor (non-gelling) carrageenan has two galactose units, whereas the transformed (gelling) carrageenan has one galactose and one 3,6-Angal unit. Therefore, 3,6-Angal is a unique indicator for the gelling units present. The sugar composition of the CRP was analyzed through hydrolysis of the carrageenan and derivatization of the sugars into alditol acetates, following a reductive hydrolysis method described by Stevenson and Furneaux [21]. Gas chromatography was carried out on an Agilent 7890B system (Agilent Technologies, Santa Clara, CA, USA) equipped with a SP2330 capillary column with a 0.25 mm internal diameter, 15 m in length, and a film thickness (d_f_) of 0.20 μm (Supelco, Bellefonte, PA, USA). A 1 μL aliquot of the sample was injected in the hot inlet operating at 260 °C and a 1:10 split ratio. Helium was used as a carrier at constant flow of 1 mL/min. The oven was programmed from 200 to 230 °C at a rate of 1 °C/min, followed by a second ramp of 20 °C/min until a final temperature of 270 °C (held for 2 min). The flame ionization was operated at 260 °C and 20 Hz. Quantification of the sugar concentration was conducted using inositol as an internal standard compared to the external standards of the sugars analyzed. The sugar analysis was carried out in triplicate from the pooled sample (*n* = 3).

#### 2.8.2. Sulfate Analysis

The sulfate analysis was analyzed as an indicator for the presence of precursor units in the carrageenan population. The precursors all have one additional sulfate than its transformed unit. A carrageenan sample with high sulfate groups indicate abundance in the precursor units. For the sulfate analysis, the CRP was first hydrolyzed following a method described by Jol et al. [22]. The resulting solution was filtered with a 0.45 μm Minisart syringe filter (Sartorius, Göttingen, Germany) and its sulfate content was analyzed using ion chromatography on a Dionex ICS 5000 system. A Dionex ionpac AS14A analytical column with an AG14A guard column and an anion self-regenerating suppressor ASR300 were used. The mobile phase consisted of 8 mM Na_2_CO_3_/1 mM NaHCO_3_ with a flow rate of 1 mL/min. A Dionex CD25 conductivity detector was used for detection. Sulfate was identified against authentic standards. The sulfate analysis was carried out in triplicate from the pooled sample (*n* = 3).

#### 2.8.3. Cation Analysis

Cations were analyzed to evaluate the nature and amount of the major counterions to the sulfate groups of the carrageenan. The analysis was carried out by first determining the ash content of the CRP by incineration at 550 °C for 5 h. The ash was then solubilized and diluted with deionized water. The solution was filtered with a 0.45 μm Minisart syringe filter (Sartorius, Göttingen, Germany) and analyzed for its cation composition on a Dionex ICS 5000 system (Thermo Fisher Scientific, Waltham, MA, USA). A Dionex ionpac CS12A cation column with a CG12A guard column and a cation self-regenerating suppressor CSRS-Ultra II 50 mm were used. The mobile phase consisted of 20 mM methanesulfonic acid (MSA) with a flow rate of 1 mL/min. A Dionex CD25 conductivity detector was used for detection. Cations were identified against authentic standards. The cation analysis was carried out in triplicate from the pooled sample (*n* = 3).

#### 2.8.4. Nitrogen Analysis

The total nitrogen analysis of the CRP was carried out by combustion on a LECO TruMac CN Analyzer (Leco Corporation, St. Joseph, MI, USA), using a nitrogen to protein conversion factor of 5 [23]. The nitrogen analysis was carried out in triplicate from the pooled sample (*n* = 3).

### 2.9. Statistical Analysis

Data were analyzed by one-way ANOVA with the Minitab statistical software (State College, PA, USA). Significant differences were determined using Tukey’s test at a level of *p* < 0.05.

## 3. Results and Discussion

The original seaweed chips were mechanically disintegrated by milling and sieved into eight different fractions: <50, 50–100, 100–150, 150–250, 250–500, 500–1000, 1000–2000, and >2000 μm. The analysis of the major components showed that there were no differences in composition (sugars, proteins, sulfate, cations) between the different size fractions as a result of milling (Table S1). This was important in order to confirm that milling did not result in an accumulation of a specific component, which may bias the result of the carrageenan extraction yield.

### 3.1. SEM Images of the Different Seaweed Size Fractions

**Figure 1:**
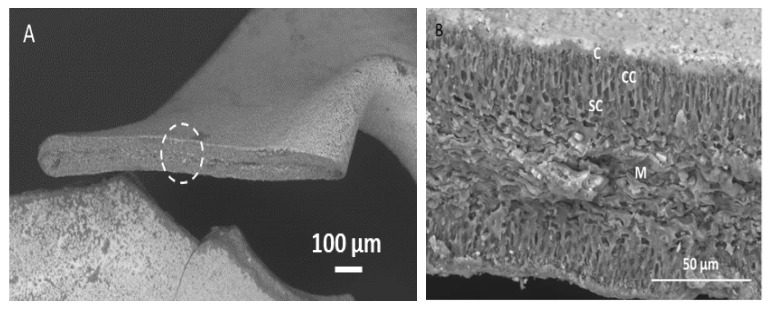
(**A**) SEM image of dried gametophyte *Chondrus crispus*. The white dashed circle shows the area taken for magnification. (**B**) Magnified cross section of the seaweed tissue. C: Cuticle; CC: Cortical cells; SC: Sub-cortical cells; M: Medullar cells.

The microstructure of the different size fractions was analyzed by SEM. The cross-sectional area of the largest size fraction (>2000 μm) (Figure 1A) showed that gametophyte *Chondrus crispus* is composed of distinct tissue morphologies (Figure 1B). Similarly to the carrageenophyte *Chondracanthus teedei* [7], the tissue morphology of gametophyte *Chondrus crispus* can be described as follows: The external part of the seaweed is covered by a cuticular layer with crystal-like structures embedded on the surface. Beneath the cuticle layer are the cortical cells, a region with packed elongated cells. Directly below the cortical cells is the subcortical cell region, where cells are more spherical, shorter in length, and surrounded by thick cell walls. The medullar region is in the center of the seaweed cross section. Here, the cells are more elongated with filamentous dimensions.

**Figure 2:**
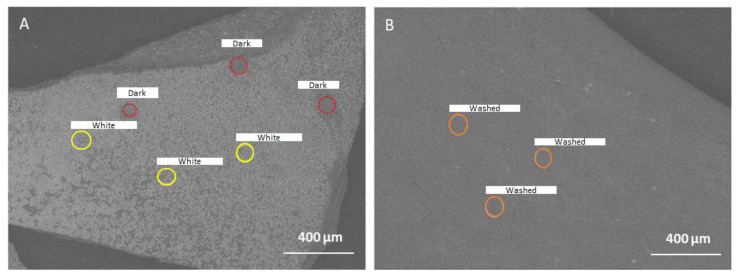
SEM image of the seaweed surface of (**A**) initial gametophyte *Chondrus crispus* chips and (**B**) gametophyte *Chondrus crispus* after washing and drying. The circles indicate the region where energy dispersive X-ray (EDX) spectroscopy was performed. ‘White’ indicates the analysis where salt crystals were present, and ‘Dark’ indicates the analysis where salt crystals were not present. ‘Washed’ indicates the analysis on the surface of the seaweed that was washed and then dried. The yellow and red circles only show the different categories of the measured area (“White” vs. “Dark” areas).

The SEM image of the seaweed surface (Figure 2A) showed that it is dotted with white specks, corresponding to the crystal-like structures mentioned above. A closer examination by the EDX analysis revealed that these white areas are rich in Na and Cl, suggesting sodium salt crystals (Table 1). The darker areas (not covered by salt) have less Na and Cl elements, and higher C, O, N, and S elements, which is more typical for a cuticular layer. Washing the seaweed (room temperature for 2 h) and drying it again was able to remove the surface NaCl crystals (Figure 2B). Moreover, the EDX analysis of the washed seaweed showed a decreased percentage of N, while S and K elements increased (Table 1). The NaCl salt on the surface of the seaweed may perhaps only be a residue of the seawater. A previous study [19] reported that the main cations detected in the wash filtrate from *Chondrus crispus* are sodium and potassium. The origin of the sodium salts could be from this external surface, whereas the potassium cations are likely to originate from the inside of the tissue, as K was not highly detected in the surface layer.

**Figure 3:**
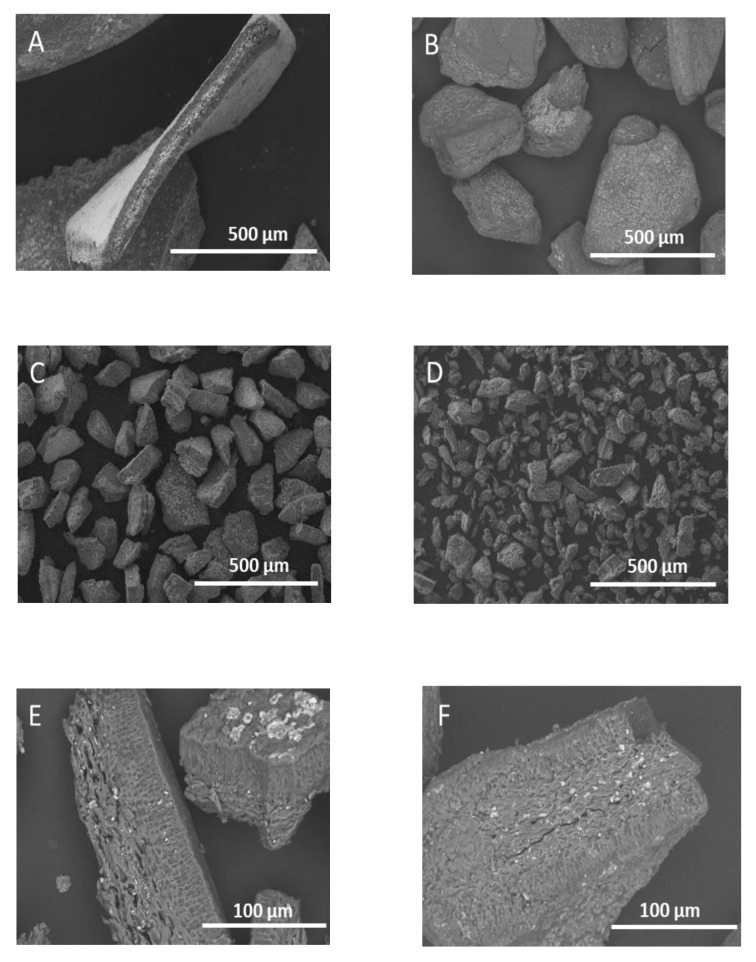
SEM image of the different size fractions of gametophyte *Chondrus crispus* that was obtained through milling and sieving. (**A**) 1000–2000 μm, (**B**) 250–500 μm, (**C**) 100–150 μm, (**D**) <50 μm. Visualization of different types of tissue breakage due to milling is shown in (**E**) along the medullar region (50–100 μm) and (**F**) perpendicular to the surface (150–250 μm).

Milling of the seaweed resulted in smaller seaweed particle sizes, with irregular shapes and non-uniform sizes (Figure 3A–D). This was visible especially in the fraction obtained on the sieve with the smallest pores, the size fraction <50 μm (Figure 3D). Milling of the seaweed caused different types of breakage of the seaweed tissue. A few tissues are broken along the medullar region (Figure 3E), while others are cut perpendicular to the surface (Figure 3F). More importantly, it was clear that the non-cuticular surface area increased with the decreasing particle sizes, exposing the cells directly to the extraction solvent (water).

### 3.2. Particle Size Distribution and Surface Area Analysis of the Seaweed Size Fractions

**Figure 4:**
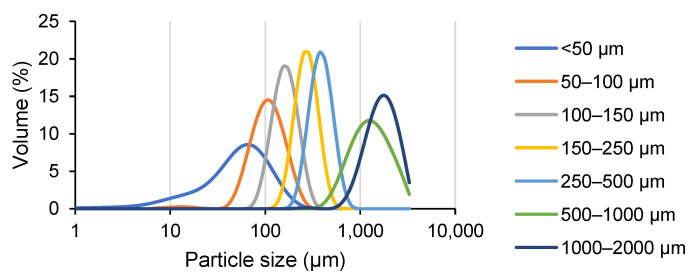
Particle size distribution analysis of the various milled gametophyte *Chondrus crispus* seaweed size fractions. The graph is an average from two measurements.

The particle size analysis and specific surface area measurements were carried out on each size fraction. The size fraction >2000 μm could not give an accurate measurement since its particle size was larger than the maximum detectable size of the particle size analyzer. Figure 4 shows that the median particle size Dv(50) decreased with the decreasing size fractions, with each fraction having a normal unimodal distribution. Since the dimension of individual red seaweed cells ranges from around 2–20 μm [7], only the smallest size fraction (<50 μm) could at least partly consist of open cells, while the other size fractions are still mainly composed of multicellular tissue fragments.

Measurement of the particle diameter showed that each size fraction contains particle sizes larger than what was expected based on the pore size of the sieves that were used (Table 2). This was reasonable, as the narrow and long particles were able to pass the sieve via its shortest dimension. However, the laser in the particle size analyzer could diffract light from the longer dimension of the particle and assimilate it as a sphere, thus resulting in an overestimation of the particle sizes. The BET specific surface area generally increased along with the decreasing particle size, until the size fraction of <50 μm, in which its specific surface area (1.03 ± 0.01 m^2^/g) increased 3-fold compared to the previous size fraction 50–100 μm (0.38 ± 0.01 m^2^/g).

### 3.3. Characteristics and Color of the Extraction Filtrate

**Figure 5:**
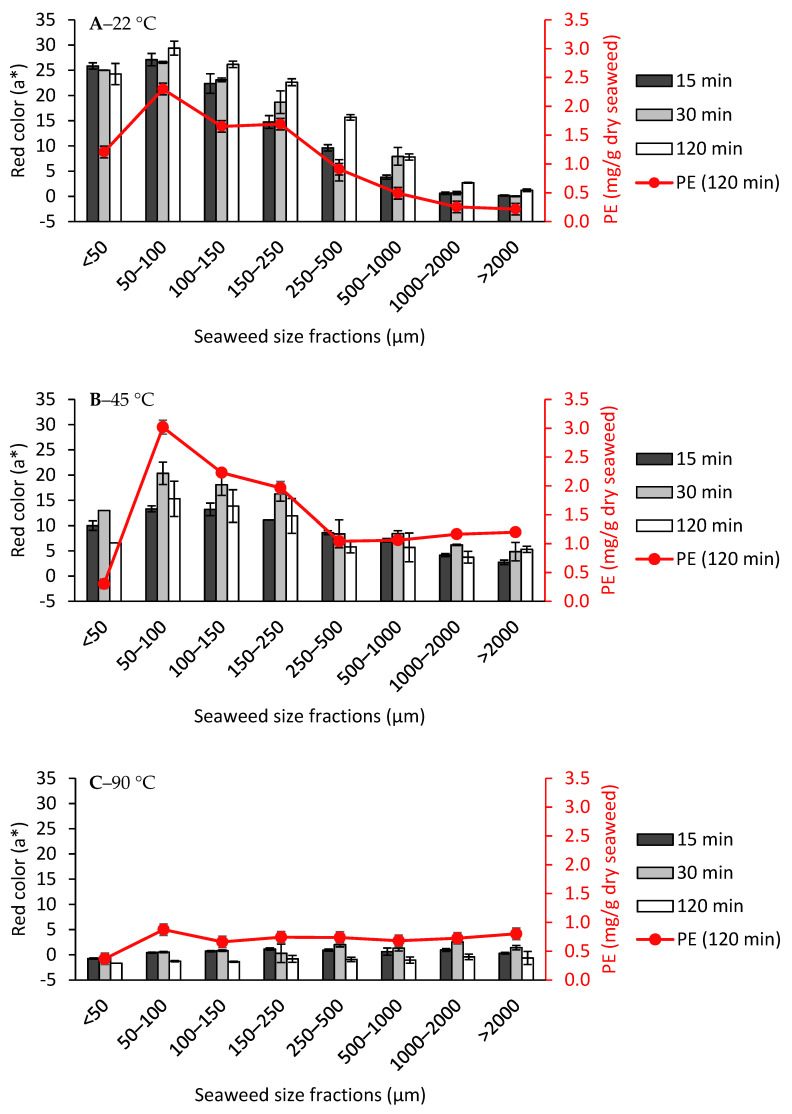
Red color values (a*) of filtrates after carrageenan extraction from each gametophyte *Chondrus crispus* seaweed size fraction at (**A**) 22 °C, (**B**) 45 °C, and (**C**) 90 °C. PE: Phycoerythrin. Values are mean ± standard deviation, *n* = 2.

The conductivity and pH values of the filtrates from all of the extractions were relatively equal, between 4535.0–5470.0 μS/cm and 6.4–6.9 respectively (Table S2). This high level of conductivity indicated the migration of salts from the seaweed into the filtrate. Depending on the extraction temperature and particle size, some of the filtrates were colored red. The redness of the filtrate was quantified by the a* value in the CIELAB space (Figure 5) and correlated with its PE concentration.

When the extractions were performed at 22 °C (Figure 5A), the larger size seaweed fractions (>1000 μm) did not result in a red colored filtrate. However, the filtrates started to be visibly red from the size fraction 500–1000 μm and below, with its a* value increasing substantially with the decreasing size of the fraction, reaching the highest a* value at the second smallest size fraction, 50–100 μm for 2 h extraction (29.4 ± 1.4). The a* value at the smallest size fraction (<50 μm) was slightly less (24.3 ± 2.1). To be more accurate, the PE concentration was also quantified. Moreover, the PE concentration showed a steady increase with the decreasing size fraction, reaching the highest concentration at 50–100 μm with a PE concentration of 2.3 ± 0.1 mg/g dry seaweed (Figure 5A), but decreased at the smallest size fraction <50 μm (1.2 ± 0.1). One reason could be that the smallest cellular debris due to the milling are accumulated in this fraction. During milling, heat is also generated due to friction and the PE in the smallest particles could already be denatured by heat. Therefore, its biomass does not contribute to PE extraction. This hypothesis is supported by the fact that the protein content in <50 μm is lower than 50–100 μm (Table S1).

The PE concentration obtained from 50–100 μm was similar to the concentration of PE found in *Mastocarpus Stellatus* (2.0 mg/g dry seaweed) [24]. Repetitive extractions would be needed to determine the total concentration of PE in the *Chondrus crispus*. However, it could be concluded that PE extraction is surface area dependent. As big size fractions are still enveloped largely by the cuticle layer, it could be that the migration of the PE was hindered by the cuticles. This could explain why a certain particle size reduction in order to expose the non-cuticular surface contact with water was needed for a certain amount of PE, which resulted in a filtrate that is visibly red colored. For each size fraction, increasing the extraction time from 15 to 120 min led to a slight, non-significant increase in redness values.

**Figure 6:**
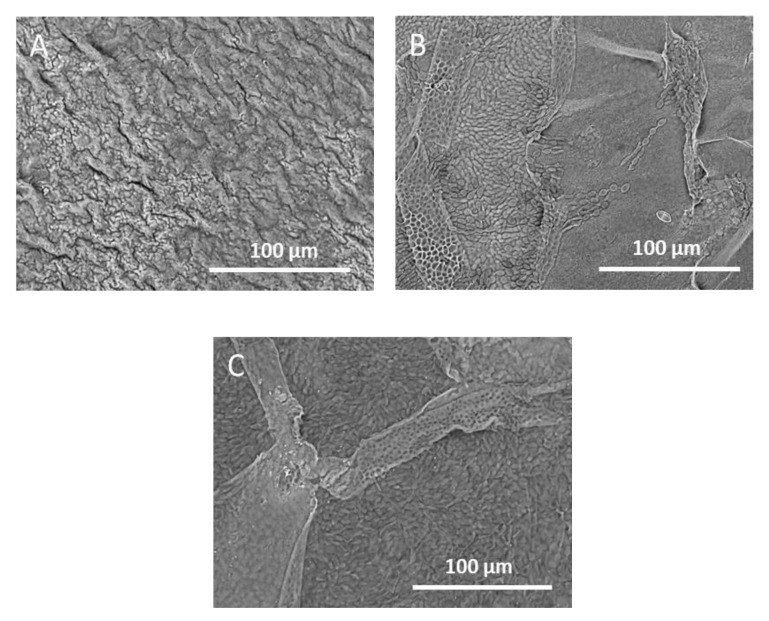
SEM image on the surface of a residual gametophyte *Chondrus crispus* from the fraction size >1000 μm after extraction at (**A**) 22 °C, (**B**) 45 °C, and (**C**) 90 °C for 2 h. The seaweed chip is left to dry at room temperature for 24 h after the extraction, before undergoing the SEM analysis. What is likely to be the cuticle layer seems intact after the extraction at 22 °C, but it is visibly peeling at the extraction of 45 and 90 °C, showing the seaweed tissue cells beneath.

Generally, extractions at 45 °C resulted in darker colored filtrates, which contained higher PE concentration compared to the filtrates extracted at 22 °C (Figure 5B) for most of the size fractions. This time, the bigger seaweed size fractions (>1000 μm) also experienced phycoerythrin diffusion, as shown by the increase in a* value (5.3 ± 0.6) and PE concentration (1.2 ± 0.1 mg/g dry seaweed) (Figure 5B) compared to when they were extracted at 22 °C, where both a* and PE concentration were close to zero (Figure 5A). The SEM analysis on the seaweed residue (>1000 μm) showed a peeling off the outermost layer of the seaweed (Figure 6A–C). This layer is likely to be the cuticle layer as the seaweed cells beneath it were exposed after the heat treatment. This direct exposure of the tissue cells to the water solvent could be a reason why PE diffusion from *Chondrus crispus* could occur from the bigger size fractions. For the size fractions <1000 μm, it was observed that despite having a higher PE concentration, their red a* values were lower compared to the extraction filtrates at 22 °C. The functionality of the PE extracted at 45 °C should be taken into consideration, as the effect of heat could already affect its color quality. Another reason could be due to an equally high concentration of phycocyanin and allophycocyanin found in the filtrates after extraction at 45 °C, which was not the case as in the filtrates after extraction at 22 °C (data not shown). In this case, the blue and red color from the phycocyanin and phycoerythrin may cause the resulting filtrate to be darker in color, thus giving a lower a* value.

At 90 °C (Figure 5C), the a* values as well as the PE concentration across all of the size fractions and extraction times were close to zero, inferring that all of the PE has denatured and lost its color. PE quickly loses its color after heating above 60 °C for 1 h [25,26].

From the results above, it can be concluded that PE extraction is specific surface area dependent, and its migration occurs readily at room temperature. When red seaweed undergoes extraction at an increased temperature, the cuticular layer starts to peel off, resulting in increased PE diffusion. However, thermal degradation of PE also occurs. Phycobiliprotein extraction should then be facilitated by keeping the extraction at lower temperatures (22 °C or lower) and with a smaller starting seaweed particle size, as normally done by grinding the seaweed in liquid nitrogen to ensure destruction of the cell walls [27,28]. Considering that the total protein content in the dry *Chondrus crispus* chips is around 16–18 wt% (Table S1), the analyzed phycoerythrin (2.3 ± 0.1 mg/g dry seaweed) only makes up a minor fraction (around 1 wt%) of the total protein content of the seaweed.

### 3.4. Carrageenan-Rich Precipitate Yield

**Figure 7:**
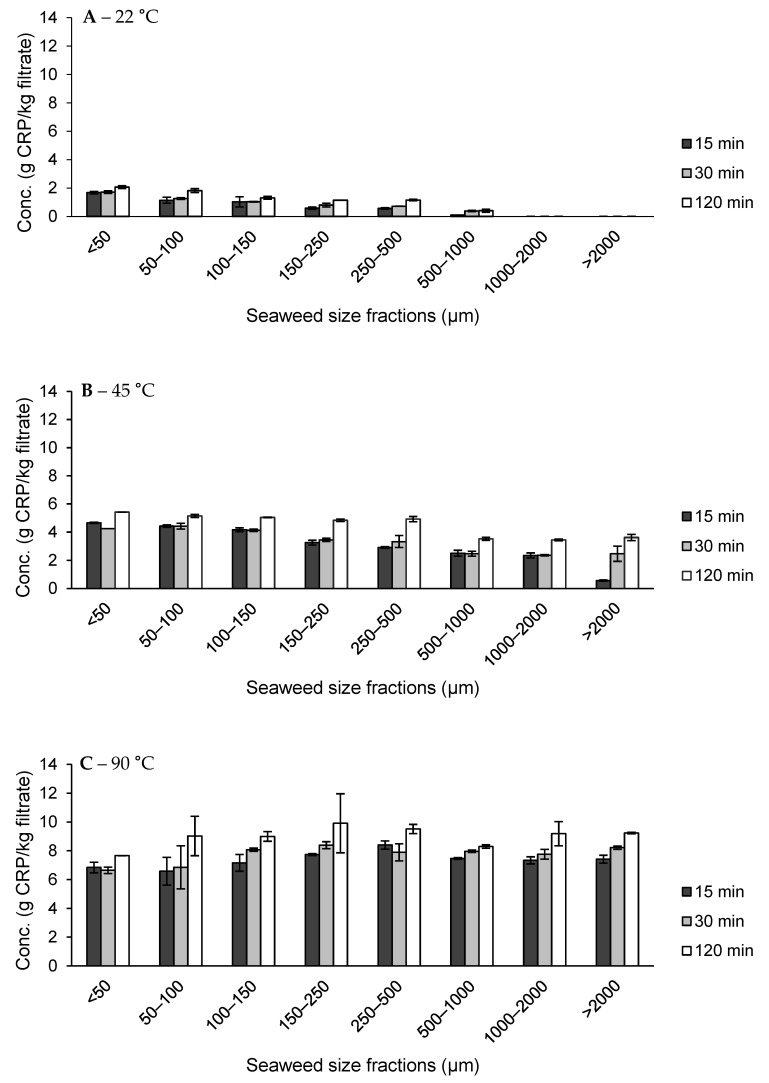
Carrageenan-rich precipitate (CRP) yield from carrageenan extraction from each gametophyte *Chondrus crispus* seaweed size fractions at (**A**) 22 °C, (**B**) 45 °C, and (**C**) 90 °C. Values are mean ± standard deviation, *n* = 2.

The carrageenan present in the filtrates was precipitated via IPA precipitation. Figure 7 showed the results of the carrageenan-rich precipitate (CRP) from the different size fractions and temperature-time combinations. The composition of some representative CRPs under different extraction conditions can be found in Table 3.

For extractions at 22 °C (Figure 7A), it was observed that CRP was not recoverable from the larger size fractions (>1000 µm). As the size of the fractions decreased, or rather, as the non-cuticular surface area increased, there was also a small steady increase in CRP yield. The maximum CRP yield was obtained at the smallest size fraction (<50 μm), for 120 min extraction (2.1 ± 0.1 g/kg). This CRP yield was still very low, indicating that most of the hybrid carrageenan could not leach out at 22 °C, regardless of the increase in non-cuticular specific surface area. The composition from this precipitate across the different extraction time and size fractions showed low carrageenan concentration (sum of galactose and 3,6-anhydrogalactose only accounting up to around 30–40 wt% of CRP) and a high amount of protein (14.1–23.5 wt% of CRP). Additional research is needed to determine where this protein originated from. As the extraction filtrate prior to IPA precipitation was high in PE concentration, it is likely that it coprecipitated with the carrageenan. Another reason could be that the carrageenan which was recovered at this level came from the protein rich cuticular region. The sulfate and potassium content are generally higher for CRP, which is extracted at 22 °C, compared to 45 and 90 °C. As it is generally accepted that the potassium ion acts as counterion to the negatively charged sulfate groups of carrageenan, more research is needed to determine whether there is a relationship between the higher amount of potassium and the type of carrageenan extracted at 22 °C. No other major amounts of cations were detected in the CRP.

At an extraction temperature of 45 °C, CRP yields were significantly higher compared to the extractions at room temperature (Figure 7B). As the size fraction decreased, there was also a small increase in CRP yield when extracting at 45 °C. The maximum yield at 45 °C (5.4 ± 0.1 g/kg) was obtained for the size fraction <50 μm extracted for 120 min. This is more than twice the amount obtained at 22 °C. Under the influence of heat, CRP was also recovered from the extraction of the large size fractions (>1000 μm). The carrageenan concentration across the extractions from different time and size fractions was higher than the CRP at 22 °C (around 45–50 wt% of CRP), while the protein content at 45 °C extractions (5.0–7.0 wt% of CRP) decreased significantly from CRP extractions at 22 °C (14.1–23.5 wt% of CRP). Additional research is needed to find the origin of these protein sources. One explanation could be that at 22 °C, due to the high PE content, PE co-precipitated with the CRP, whereas these bonds are broken due to the increasing temperature.

The CRP yields were highest at the extraction temperature of 90 °C (between 8.3 ± 0.1 to 9.9 ± 2.1 g/kg filtrate), almost twice the amount obtained at 45 °C (Figure 7C). At this high temperature, the CRP yield was not dependent on the size of the fractions. During the extraction of the smaller size fractions (<100 μm), it was observed that the seaweed powders were aggregated during extraction, hindering the separation between the hybrid carrageenan and the residue. This probably explains the smaller CRP yield obtained for the smaller size fractions. The carrageenan concentration was highest at 90 °C compared to the other extraction temperatures (around 52–67 wt% of CRP). Accordingly, the protein content was lowest at this extraction temperature as well (4.4–5.9 wt% of CRP).

From these results, it can be concluded that the total obtainable CRP at low temperatures (22 and 45 °C) was negligible compared to the extraction at 90 °C, even at a reduced particle size. At room temperature, even with substantially more non-cuticular surface area for the smallest size fractions, hybrid carrageenan could still not leach out effectively. The reduction of particle size coupled with the increased surface area could not replace the effect of heat for hybrid carrageenan extraction. Therefore, hybrid carrageenan extraction is temperature dependent and less dependent on the specific surface area. This resistance seems to stem from the native structure of the hybrid carrageenan itself in the seaweed matrix, which is very likely to be in the helix structure. Heat is then needed to dissociate the helical structure, turning it into a coil structure, and allowing it to be hydrated/solubilized and thus, recovered. The composition of the carrageenan in the CRP was not significantly different across the different extraction temperatures, except for the fact that the protein concentration was significantly higher when extracting at 22 °C. Determining the origin of this protein could help in better understanding the interactions between carrageenan and proteins.

## 4. Conclusions

The particle size reduction of gametophyte *Chondrus crispus* seaweed, which caused an increase in the specific surface area, did not lead to a substantial increase of hybrid carrageenan extraction at mild temperatures. Solubilization of the polymer in the cell wall matrix, most likely requiring the helix to coil transition, is a prerequisite for hybrid carrageenan extraction, which is usually achieved using heat. The low CRP yield that was obtained at 22 °C extraction from the smaller size fractions resulted in CRF samples relatively high in proteins. The particle size reduction did lead to a significant increase in the redness of the filtrate, which corresponds to the presence of PE. The redness value of the filtrate was highest at the lowest extraction temperature tested (22 °C) and for the smallest size fraction (50–100 μm). Phycobiliproteins seem to be able to diffuse freely out of the seaweed, as long as there is sufficient disintegration to the seaweed tissue, even at 22 °C. At this level, the carrageenan yield is not compromised as they are still embedded in the cell wall matrix. Therefore, the mechanisms of migration of the red pigments and hybrid carrageenan appear to be different. With this knowledge, a strategy could be devised whereby red pigments are harvested first under mild temperature conditions, followed by carrageenan extraction under more intense extraction conditions. In the future, exact quantification and purification of the PE from these filtrates would be needed to evaluate the economic feasibility of this type of multi-step extraction. The functionality of the resulting hybrid carrageenan from this multi-step extraction should also be compared to the existing standards. Finally, the seaweed residue after phycoerythrin and carrageenan extraction could potentially be further valorized amongst the other bioethanol or feed applications [29,30,31].

## Figures and Tables

**Table 1:** Elemental mass composition determined by energy dispersive X-ray (EDX) spectroscopy on the various surface areas (cuticle) of the gametophyte *Chondrus crispus* seaweed (Figure 2).

	Composition (wt%)								
	N	S	Na	Cl	K	Ca	Mg	C	O
Initial-white	N.D.	2.8 ± 1.0 ^b^	24.8 ± 1.0 ^a^	35.9 ± 11.5 ^a^	1.1 ± 0.5 ^b^	0.8 ± 0.1 ^ab^	n.d.	27.1 ± 9.4 ^a^	7.3 ± 4.3 ^b^
Initial-dark	9.0 ± 2.7 ^a^	4.9 ± 0.8 ^b^	3.2 ± 0.8 ^b^	4.4 ± 0.5 ^b^	2.4 ± 0.9 ^b^	0.6 ± 0.1 ^ab^	1.2 ± 0.1 ^a^	40.9 ± 1.3 ^a^	33.2 ± 0.6 ^a^
Washed	3.8 ± 0.6 ^b^	11.7 ± 0.8 ^a^	3.4 ± 0.8 ^b^	1.4 ± 0.2 ^b^	5.2 ± 0.6 ^a^	1.1 ± 0.1 ^a^	1.8 ± 0.3 ^a^	33.4 ± 4.1 ^a^	35.1 ± 2.5 ^a^

Values are mean ± standard deviation, *n* = 3. N.D.: Not detected. Values that share the same letter within a category are not significantly different (*p* < 0.05).

**Table 2:** Particle size characteristics and BET surface area of each gametophyte *Chondrus crispus* size fraction. Dv(10), Dv(50), and Dv(90) refer to the particle size diameter values where 10%, 50%, and 90% of the sample volume (starting from the smallest particles) are contained, respectively. D(4,3) refers to the volume mean particle size diameter.

Fraction Size (μm)	Diameter (μm)				BET Surface Area (m^2^/g)
	Dv(10)	Dv(50)	Dv(90)	D(4,3)	
<50	14.9 ± 6.3	54.3 ± 7.2	123.5 ± 4.9	63.2 ± 6.5	1.03 ± 0.01
50–100	62.3 ± 0.1	106 ± 1.4	177.5 ± 3.5	113.5 ± 2.1	0.38 ± 0.01
100–150	108 ± 1.4	161 ± 0.1	237 ± 1.4	166.5 ± 2.1	0.31 ± 0.01
150–250	191 ± 21.2	270.5 ± 29.0	381 ± 42.4	280 ± 29.7	0.22 ± 0.01
250–500	268.5 ± 3.5	384.5 ± 3.5	552.5 ± 4.9	385 ± 22.6	0.23 ± 0.01
500–1000	676.5 ± 40.3	1265 ± 49.5	2360 ± 28.3	1400 ± 42.4	0.05 ± 0.01
1000–2000	996 ± 5.7	1685 ± 7.1	2665 ± 35.4	1760 ± 14.1	0.04 ± 0.01

Values are mean ± standard deviation, *n* = 2.

**Table 3:** Compositional analysis of carrageenan-rich precipitate obtained at different extraction conditions and from small (<50 μm) and large (500–1000 μm) size fractions.

Extraction Conditions			Major Composition of CRP (wt%)				
T (°C)	t (min)	Size Fraction (μm)	Galactose	3,6-Angal	Sulfate	Protein	Potassium
22	15	<50	28.6 ± 2.0 ^d^	12.1 ± 1.0 ^d^	28.8 ± 1.8 ^bc^	15.7 ± 1.0 ^b^	12.2 ± 0.8 ^cd^
	120	<50	27.9 ± 1.4 ^d^	11.3 ± 0.7 ^d^	31.5 ± 0.2 ^ab^	14.1 ± 0.3 ^c^	13.1 ± 0.1 ^bc^
		500–1000	19.3 ± 0.6 ^e^	9.7 ± 0.3 ^d^	28.5 ± 0.7 ^bc^	23.5 ± 0.6 ^a^	16.7 ± 0.4 ^a^
45	15	<50	27.9 ± 0.1 ^d^	23.7 ± 2.0 ^a^	28.0 ± 0.2 ^c^	7.0 ± 0.1 ^d^	11.6 ± 0.1 ^d^
		500–1000	28.1 ± 0.1 ^d^	18.3 ± 1.0 ^bc^	32.4 ± 0.2 ^a^	6.8 ± 0.3 ^d^	13.5 ± 0.1 ^b^
	120	<50	34.9 ± 1.5 ^bc^	18.1 ± 0.6 ^bc^	26.7 ± 0.8 ^c^	6.1 ± 0.3 ^de^	9.6 ± 0.3 ^e^
		500–1000	35.1 ± 1.6 ^bc^	18.2 ± 1.4 ^bc^	28.4 ± 2.2 ^bc^	5.0 ± 0.2 ^ef^	9.7 ± 0.7 ^e^
90	15	<50	35.3 ± 1.8 ^bc^	17.7 ± 0.6 ^c^	27.0 ± 1.4 ^c^	5.9 ± 0.4 ^de^	9.5 ± 0.5 ^e^
		500–1000	37.2 ± 2.1 ^b^	21.2 ± 1.7 ^ab^	26.2 ± 1.8 ^c^	4.4 ± 0.4 ^f^	7.8 ± 0.5 ^f^
	120	<50	33.2 ± 0.5 ^c^	19.0 ± 0.4 ^bc^	28.7 ± 0.2 ^bc^	5.5 ± 0.1 ^ef^	8.9 ± 0.1 ^ef^
		500–1000	44.6 ± 1.0 ^a^	22.4 ± 0.7 ^a^	19.9 ± 0.3 ^d^	5.1 ± 0.3 ^ef^	5.0 ± 0.1 ^g^
Kappa Reference			36.3 ± 1.3	36.3 ± 1.4	17.1 ± 0.6	TR	10.0 ± 0.3

Values are mean ± standard deviation, *n* = 3. TR = Trace (<1 wt%). Values that share the same letter within a category are not significantly different (*p* < 0.05).

## Data Availability

All the data are included in the article and Supplementary Materials.

## Supplementary Materials

The following are available online at https://www.mdpi.com/article/10.3390/foods10122928/s1. Table S1: Composition of the different seaweed size fractions; Table S2: Filtrate conductivity and pH values from carrageenan extraction of each size fraction at various extraction times and temperatures.
