# Supplementary material for: Mechanical Disintegration and Particle Size Sieving of Chondrus crispus (Irish Moss) Gametophytes and Their Effect on Carrageenan and Phycoerythrin Extraction
Source: Foods. 2021 Nov 26;10(12):2928. doi: 10.3390/foods10122928 (PMC8701067; doi:10.3390/foods10122928)
Supplement: Supplementary file 1 [file foods-10-02928-s001.zip › Supplementary-R.pdf]

Supplementary Table S1. Composition of the different seaweed size fractions.

Size fractions (μm)	Seaweed composition (wt%)							
	3,6-Angal	Galactose	Xylose	Glucose	Protein	Sodium	Potassium	Sulfate
<50	12.8 ± 0.7	24.7 ± 0.4	0.6 ± 0.1	1.3 ± 0.1	17.0 ± 0.1	7.5 ± 0.1	9.1 ± 0.1	13.6 ± 0.1
50-100	11.6 ± 2.2	23.0 ± 3.3	0.5 ± 0.2	1.2 ± 0.1	18.3 ± 0.1	5.5 ± 0.1	7.3 ± 0.1	12.9 ± 0.1
100-150	11.6 ± 2.4	22.6 ± 2.6	0.5 ± 0.1	1.0 ± 0.1	18.2 ± 0.1	6.2 ± 0.1	7.2 ± 0.1	13.1 ± 0.1
150-250	11.7 ± 1.9	22.1 ± 0.4	0.5 ± 0.1	0.9 ± 0.1	17.7 ± 0.1	5.5 ± 0.1	6.2 ± 0.1	17.4 ± 0.1
250-500	14.3 ± 3.6	26.1 ± 2.3	0.5 ± 0.1	1.2 ± 0.1	16.9 ± 0.1	6.7 ± 0.1	7.7 ± 0.1	13.7 ± 0.1
500-1000	12.9 ± 0.9	25.5 ± 1.3	0.4 ± 0.1	1.0 ± 0.2	18.1 ± 0.3	6.2 ± 0.1	6.6 ± 0.1	13.2 ± 0.1
1000-2000	15.2 ± 3.2	28.9 ± 3.1	0.7 ± 0.1	1.9 ± 0.1	17.7 ± 0.6	6.9 ± 0.1	7.2 ± 0.1	12.4 ± 0.1
>2000	16.6 ± 5.7	26.9 ± 4.7	0.7 ± 0.1	0.7 ± 0.1	16.1 ± 0.1	6.6 ± 0.1	7.4 ± 0.1	14.7 ± 0.1

Values are mean ± standard deviation, n = 2. 3,6-Angal = 3,6-Anhydrogalactose

Supplementary Table S2. Filtrate conductivity and pH values of filtrates extracted from each size fractions at various extraction times and temperatures.

T (°C)	Fraction size (µm)	Conductivity (µS/cm)			pH		
		15 min	30 min	120 min	15 min	30 min	120 min
22	<50	5000 ± 99	5010 ± 56	5235 ± 63	6.9 ± 0.1	6.9 ± 0.1	6.9 ± 0.1
	50-100	4770 ± 70	4695 ± 21	4875 ± 304	6.8 ± 0.1	6.8 ± 0.1	6.8 ± 0.1
	100-150	4675 ± 63	4535 ± 35	4785 ± 7	6.7 ± 0.1	6.8 ± 0.1	6.8 ± 0.1
	150-250	4755 ± 120	4585 ± 63	4940 ± 70	6.7 ± 0.1	6.7 ± 0.1	6.7 ± 0.1
	250-500	4620 ± 42	4610 ± 113	4645 ± 91	6.7 ± 0.1	6.7 ± 0.1	6.7 ± 0.1
	500-1000	4650 ± 28	4625 ± 63	4605 ± 21	6.8 ± 0.1	6.8 ± 0.1	6.9 ± 0.1
	1000-2000	4750 ± 56	4775 ± 91	4745 ± 77	6.7 ± 0.1	6.7 ± 0.1	6.7 ± 0.1
	>2000	4715 ± 49	4725 ± 21	4760 ± 183	6.8 ± 0.1	6.7 ± 0.1	6.8 ± 0.1
45	<50	5365 ± 636	5630 ± 50	5080 ± 50	6.8 ± 0.1	6.8 ± 0.1	6.9 ± 0.1
	50-100	5035 ± 134	5050 ± 11	5090 ± 56	6.6 ± 0.1	6.6 ± 0.1	6.7 ± 0.1
	100-150	5130 ± 311	5015 ± 77	5065 ± 120	6.6 ± 0.1	6.6 ± 0.1	6.6 ± 0.1
	150-250	4795 ± 374	5050 ± 14	5220 ± 56	6.6 ± 0.1	6.6 ± 0.1	6.5 ± 0.1
	250-500	4810 ± 42	5245 ± 431	5385 ± 134	6.6 ± 0.1	6.6 ± 0.1	6.6 ± 0.1
	500-1000	4855 ± 35	5075 ± 190	5245 ± 417	6.7 ± 0.1	6.7 ± 0.1	6.7 ± 0.1
	1000-2000	5020 ± 28	5185 ± 374	5350 ± 650	6.6 ± 0.1	6.6 ± 0.1	6.6 ± 0.1
	>2000	5060 ± 14	5525 ± 21	5185 ± 459	6.6 ± 0.1	6.6 ± 0.1	6.6 ± 0.1
90	<50	5535 ± 63	5760 ± 50	5840 ± 50	6.9 ± 0.1	6.9 ± 0.2	6.9 ± 0.1
	50-100	5300 ± 134	5070 ± 113	5845 ± 56	6.8 ± 0.1	6.7 ± 0.1	6.6 ± 0.1
	100-150	5360 ± 311	5360 ± 77	5815 ± 120	6.7 ± 0.1	6.7 ± 0.1	6.5 ± 0.1
	150-250	5650 ± 374	5280 ± 14	5710 ± 56	6.6 ± 0.1	6.8 ± 0.2	6.4 ± 0.1
	250-500	5525 ± 42	5275 ± 431	5625 ± 134	6.6 ± 0.1	6.6 ± 0.1	6.5 ± 0.2
	500-1000	5470 ± 35	5415 ± 190	5345 ± 417	6.6 ± 0.1	6.7 ± 0.1	6.6 ± 0.2
	1000-2000	5430 ± 28	5460 ± 374	5650 ± 650	6.6 ± 0.1	6.6 ± 0.1	6.5 ± 0.1
	>2000	5515 ± 14	5695 ± 21	5685 ± 459	6.7 ± 0.1	6.7 ± 0.1	6.5 ± 0.1

Values are mean ± standard deviation, *n* = 2.
